# Interleukin-8 is not a predictive biomarker for the development of the acute promyelocytic leukemia differentiation syndrome

**DOI:** 10.1186/s12885-020-07330-1

**Published:** 2020-08-28

**Authors:** Luciana Yamamoto de Almeida, Diego Antonio Pereira-Martins, Ana Sílvia Gouvêa Lima, Márcia Sueli Baggio, Luisa Corrêa de Araujo Koury, Ana Paula Lange, Sarah Cristina Bassi, Priscila Santos Scheucher, Eduardo Magalhães Rego

**Affiliations:** 1 Hematology Division, Department of Medical Images, Hematology, and Clinical Oncology, University of Sao Paulo at Ribeirao Preto Medical School, Ribeirao Preto, Brazil; 2 Center for Cell Based Therapy, University of Sao Paulo at Ribeirao Preto Medical School, Ribeirao Preto, Brazil; 3 Hemostasis Laboratory, Hospital das Clínicas da Faculdade de Medicina de Ribeirão Preto, University of Sao Paulo, Ribeirao Preto, Brazil; 4 Hematology Division, LIM31, Faculdade de Medicina, University of Sao Paulo, Av Dr Eneas Carvalho de Aguiar 155, 1st Floor, Hemocentro, São Paulo, SP CEP05403-000 Brazil

**Keywords:** Acute promyelocytic leukemia, Differentiation syndrome, Interleukin-6 (IL-6), Interleukin-8 (IL-8)

## Abstract

**Background:**

Differentiation syndrome (DS) is the main life-threatening adverse event that occurs in acute promyelocytic leukemia (APL) patients treated with all-trans retinoic acid (ATRA). Cytokine imbalances have been reported to play role during the developing of acute promyelocytic leukemia differentiation syndrome (APL-DS). However, the relationship between the plasma cytokine levels and their prognostic value for the prediction of DS developing in patients with APL during the treatment with ATRA and anthracyclines has not been previously reported.

**Methods:**

In this study, we followed an APL cohort (*n* = 17) over 7 days of ATRA therapy in DS (*n* = 6) and non-DS groups (*n* = 11). Interleukin (IL)-1β, IL-6, IL-8, IL-10, IL-12p70 and TNF-α were measured in the peripheral blood plasma from 17 patients with APL and 11 healthy adult controls by using the cytometric bead array method.

**Results:**

In non-DS patients, IL-8 plasma levels were significantly reduced in the seventh day of ATRA treatment (34.16; 6.99 to 147.11 pg mL^− 1^ in D0 vs. 10.9; 0 to 26.81 pg mL^− 1^ in D7; *p* = 0.02) whereas their levels did not discriminate between DS and non-DS development during the entire induction period (all *p* > 0.05 in D0, D3, and D7). No significant differences were found in IL-6 levels between groups (*p* > 0.05 in D0-D7). Other cytokines tested were all undetectable in patients with APL or healthy controls.

**Conclusions:**

We demonstrated that the modulation of IL-8 following ATRA treatment may occur regardless of the development of DS and, therefore, does not appear to be a predictive biomarker to monitor the APL-DS.

## Background

Differentiation syndrome (DS) is a life-threatening adverse event that occurs in approximately 20–25% of patients with acute promyelocytic leukemia (APL) undergoing induction therapy with all-trans retinoic acid (ATRA) [1, 2]. During the APL-DS, changes in seric levels of cytokines [3], and in cellular adhesion/migration properties [1, 4, 5], as well as endothelial damage [6] have been reported to be related to ATRA-driven DS. The in vitro release of pro-inflammatory cytokines such as interleukin (IL)-1β, IL-6, IL-8, and tumor necrosis factor-α (TNF-α) have been reported to coincide with ATRA-induced differentiation of APL blasts, which may lead in vivo to a systemic inflammatory response syndrome (SIRS) [3, 7]. Moreover, the ATRA-differentiating APL cells have increased ability to migrate from the blood flow into the tissues by upregulating molecules involved in cell adhesion (e.g.: ICAM: Intercellular Adhesion Molecule-1 and 2) and migration (e.g.: E-selectin and β2-integrin) [5, 8].

The lung is the most clinically relevant target organ in APL-DS patients, which can develop distinct pulmonary complications [9]. Accordingly, it was experimentally demonstrated that increased expression of ICAM-1 on the lung of NOD/scid mice after ATRA therapy is important for the pulmonary infiltration of APL cells [10]. Moreover, the IL-8 secretion by A549 alveolar epithelial cells support the chemotactic transmigration of ATRA-treated NB4 APL cells toward A549 cells [11]. In fact, IL-8 plays a important role in acute inflammation by activating and chemoattracting neutrophils [12]. Patients with acute myeloid leukemia (AML) that express lower levels of IL-8 have better survival outcomes [13]. Accordingly, the IL-8 receptor CXCR2 is an adverse prognostic factor in AML and its inhibition decreases the proliferation of AML cell lines and primary samples [14].

Despite the lack of biomarkers that predict development of DS, Shibakura et al. observed that serum levels of IL-8 were increased during the course of ATRA treatment in two patients who developed APL-DS [15]. To support these in vivo findings, the same authors confirmed that the in vitro IL-8 expression was also up-regulated in leukemic primary cells from both patients with APL after incubation with ATRA [15]. Nevertheless, no previous study has investigated and compared the IL-8 plasma levels in the plasma of DS and non-DS patients with APL during the treatment with ATRA to determine whether this cytokine has the potential to predict the development of the APL-DS.

## Methods

### Patients

The patients were treated according to the International Consortium on APL protocol [16] and cytokine levels were also evaluated in the 17 patients with APL who developed (DS-group; *n* = 6) or not (non-DS group; *n* = 11) DS during the treatment with ATRA and anthracyclines. The diagnosis of DS was based on the presence of any signs and symptoms of dyspnea, renal failure, hypotension, fever, weight gain (greater than 5 Kg), edema and pulmonary congestion. Then, the clinical manifestations of DS were adequately managed following the European LeukemiaNet recommendations [17]. Samples from 11 healthy adult volunteers were used as controls.

### Cytokine quantification

Plasma samples were obtained at diagnosis (D0), after three (D3) and seven (D7) days of treatment with ATRA and anthracyclines. Briefly, after centrifugation of heparinized peripheral blood, the plasma samples were immediately aliquoted and stored at − 80 °C until the experiment was conducted. The concentrations of IL-8, IL-1β, IL-6, IL-10, IL-12p70 and TNF in the plasma of patients with APL and healthy controls were measured once after collection of each individual samples by using the cytometric bead array assay (CBA-Human Inflammatory Cytokine Kit, BD Biosciences) according to the manufacturer’s instructions. The standard curves concentrations for each cytokine ranged from 20 to 5000 pg/mL. Samples were acquired on the FACSCalibur flow cytometer (BD Biosciences) and analyzed using FCAP Array™ software (BD Biosciences).

### Statistical analyses

The plasma concentrations of each cytokine were compared among patients with APL at D0 and during the days of treatment with ATRA and anthracyclines (D3 and D7) using the Two-way ANOVA, followed by Tukey’s post-test for the comparison between –DS and non-DS groups and Friedman’s test, followed by Dunn’s multiple comparison post-test when –DS and non-DS groups were gathered together. Fisher’s two-tailed exact test (categorical variables) or Mann-Whitney U test (continuous variables) was used to assess the possible differences between IL-6 and IL-8 levels and clinical parameters such as age, gender, WBC and Plt counts, fibrinogen concentration, relapse-risk and death during induction between DS and non-DS groups. Statistical analyses were performed using SPSS software (version 19.0; IBM Corp., Armonk, NY, USA). A *p* value < 0.05 was considered significant.

## Results

Seventeen patients with APL (10 females, 7 males) with an age range of 19–72 years (median age, 36 ± 16 years) diagnosed at the Clinics Hospital of Ribeirão Preto (HCFMRP), University of São Paulo, from March 2007 through July 2013 were included in this study. All the six patients with DS presented with dyspnea, pulmonary infiltrates and unexplained fever and, in two cases there was evidence of kidney failure as well (severe DS). The symptoms started between 9th and 18th day of ATRA therapy. All but one recovered from the DS and achieved and remain in complete remission. Eight healthy controls (8 females, 3 males) were included as controls. Samples were collected only from control subjects with no history of fever within 1 week, use of any medications or drugs, pregnancy, and chronic diseases. In our cohort, IL-1β, IL-10, IL-12p70 and TNF-α were not detected in the plasma of patients with APL regardless of the development of DS. These findings may due to the absence of such cytokines in the samples or to the fact that these cytokine concentrations are below the detection limit of the CBA (IL-1β: < 7.2 pg/mL; IL-10: < 3.3 pg/mL; IL-12p70: < 1.9 pg/mL; TNF-α: < 3.7 pg/mL).

**Fig. 1:**
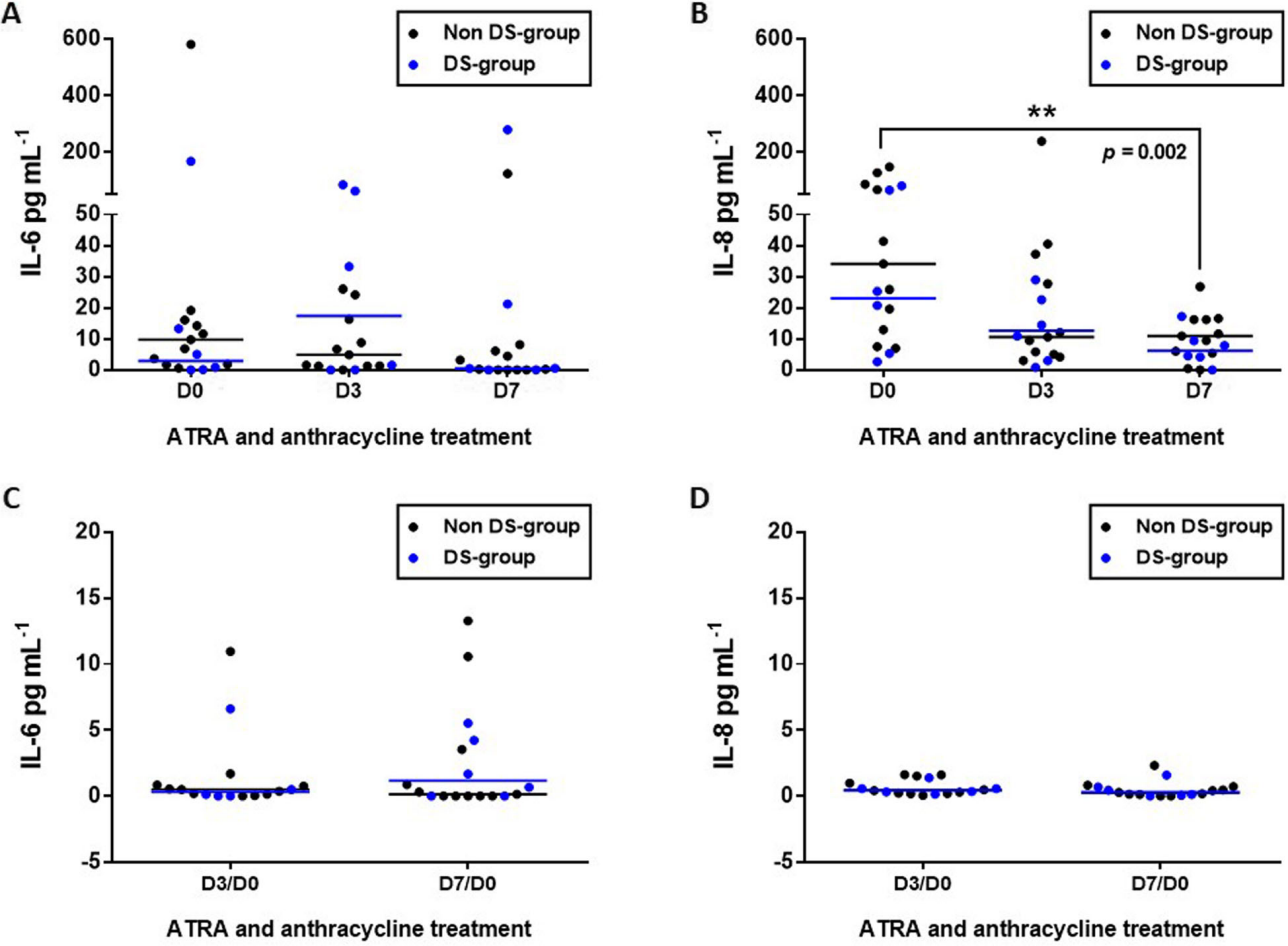
IL-6 and IL-8 plasma levels in APL-DS patients during ATRA and anthracyclines treatment. **a** The comparison of the interleukin (IL) -6 plasma levels between patients with APL treated with all-trans retinoic acid (ATRA) and anthracyclines who developed (DS-group; *n* = 6) or not (non-DS group; *n* = 11) the differentiation syndrome (DS) were unchanged. **b** ATRA and anthracyclines significantly reduced the IL-8 levels regardless of the DS development (D7 vs. D0 of DS and non-DS groups combined together, *p* = 0.002 – Friedman’s test, followed by Dunn’s multiple comparison post-test). The IL-6 (**c**) and IL-8 (**d**) D3/D0 and D7/D0 ratios did not differ between DS- and non-DS patients with APL. The horizontal lines represent the median of cytokine plasma concentration in DS- (blue) and non-DS (black) groups

Table 1 shows the levels of IL-6 and -8 at D0, after three and 7 days of treatment. IL-6 and IL-8 were not detected in healthy control group (data not shown). The distribution of the plasma concentration levels of IL-6 and IL-8 at D0 ranged from 0 to 83.74 pg mL^− 1^ and from 0.83 to 238.60 pg mL^− 1^, respectively. Although the median values of IL-6 and IL-8 were lower in the group of patients who developed DS compared to the non-DS group the differences were not significant (Fig. 1a-b; Table 1; Supplementary file 1; both *p* > 0.05). At the D3, the median value of IL-6 concentrations was higher in the DS group, but again the difference was not significant (Fig. 1a-b; Table 1; Supplementary file 1; *p* = 0.60). In contrast, at the seventh day of treatment with ATRA, we observed that APL plasma samples in non-DS group exhibited significant decreased IL-8 levels (34.16; 6.99 to 147.11 pg mL^− 1^ in D0 vs. 10.9; 0 to 26.81 pg mL^− 1^ in D7; *p* = 0.02 - Fig. 1b; Table 1; Supplementary file 1). Although there was a decrease in IL-8 levels in DS-group it was not significant (23.09; 2.63 to 79.64 pg mL^− 1^ in D0 vs. 6.2; 0 to 17.24 pg mL^− 1^ in D7; *p* = 0.06 - Fig. 1b; Table 1; Supplementary file 1). In addition, there were no differences between the two groups in the IL-6 levels throughout treatment (Fig. 1a; Table 1; Supplementary file 1; all *p* > 0.05). Of note, assessing our cohort, only 6/17 (2 from DS- and 4 from non-DS group) and 2/17 (1 from DS- and 1 from non-DS group; Supplementary file 1) patients with APL, respectively, for the levels of IL-6 and IL-8 became undetectable at D7, such as observed in healthy controls. Moreover, our study not detected significant differences between IL-6 and IL-8 levels at D0/D3 (Fig. 1c; Supplementary file 1; both *p* > 0.05) and D0/D7 (Fig. 1d; Supplementary file 1; both *p* > 0.05) ratios when comparing DS- and non-DS groups.

**Table 1:** Effects of ATRA and anthracyclines on the secretion of cytokines in APL patients with or without DS

Cytokine	All patients, (*n* = 17)	DS group, (*n* = 6)	Non-DS group, (*n* = 11)	*P*-value^1^
		Median (range)	Median (range)	
IL-6				
D0	6.86 (0.00, 580.19)	2.93 (0.00, 167.03)	9.80 (0.61, 580.19)	0.28
D3	4.92 (0.00, 83.74)	17.39 (0.00, 83.74)	4.92 (0.00, 26.04)	0.60
D7	0.48 (0.00, 279.10)	0.26 (0.00, 122.96)	0.52 (0.00, 279.10)	0.70
IL-8				
D0	25.92 (2.63, 147.11)	23.10 (2.63, 79.64)	34.16 (6.99, 147.11)	0.33
D3	10.91 (0.83, 238.60)	12.71 (0.83, 28.99)	10.62 (3.02, 238.60)	0.67
D7	9.37 (0.00, 26.81)	6.20 (0.00, 17.24)	10.91 (0.00, 26.81)	0.31

*APL* Acute promyelocytic leukemia, *DS* Differentiation syndrome

^1^Mann-Whitney U test was used to compare cytokine plasma levels between DS and non-DS groups

Table 2 shows the distribution of the main clinical and laboratory variables in the groups of patients with low or high levels of IL-6 and IL-8 at diagnosis. Patients with WBC counts higher than 10^9^/L presented lower concentrations of IL-8 at diagnosis. In fact, with the exception of WBC counts (*p* = 0.04; Table 3; Supplementary file 1), the clinical features and laboratory results of patients with APL at D0 were not associated with DS development (all *p* > 0.05; Table 3; Supplementary file 1). Patients who develop (median value 6.2 ± 5.9 pg mL^− 1^) or not (median value 10.9 ± 7.9 pg mL^− 1^) the DS persisted with detectable low plasma concentrations of IL-8 after 7 days of treatment with ATRA. Finally, the variation of IL-8 levels in patients with APL following ATRA treatment occurred independently of the development or not of DS (*p* = 0.002; D0 vs. D7 of DS- and non-DS groups combined together - Fig. 1b; Supplementary file 1).

**Table 2:** Clinical and laboratory variables in the groups of patients with low or high levels of IL-6 and IL-8 at diagnosis

	IL-6 high Median (range) [pg/mL]	R^b^	P^b^	IL-6 low Median (range) [pg/mL]	R^b^	P^b^	IL-8 high Median (range) [pg/mL]	R^b^	P^b^	IL-8 low Median (range) [pg/mL]	R^b^	P^b^
Gender^a^												
Female (*n* = 10)	13.3 (9.8–16.0)	–	–	1.2 (0–5.0)	–	–	65.8 (41.4–147.1)	–	–	12.9 (2.6–25.9)	–	–
Male (*n* = 7)	93.1 (6.8–580.1)	–	–	1.7 (0–3.6)	–	–	85.3 (34.1–285.1)	–	–	7.5 (3.3–19.6)	–	–
Age, years												
< 40 (*n* = 11)	13.3 (6.8–580.1)	0.39	0.38	2.7 (0–5.0)	- 0.20	0.80	73.0 (34.1–147.1)	0.71	0.11	19.6 (34.1–147.1)	0.40	0.50
≥ 40 (*n* = 6)	17.6 (16.0–19.2)	*c*	*c*	0.73 (0–2.9)	0.31	0.54	126.1 (41.4–285.1)	- 0.50	0.66	6.9 (2.6–20.8)	0.60	0.28
Leukocyte counts, × 10^9^/L												
< 10 (*n* = 11)	13.3 (6.8–580.1)	- 0.63	0.12	2.3 (0–5.0)	- 0.31	0.54	66.5 (34.1–285.1)	- 0.25	0.58	13.6 (3.3–25.9)	- 0.82	0.04*
≥ 10 (*n* = 6)	93.1 (19.2–167.0)	*c*	*c*	0.8 (0–1.8)	0.91	0.25	102.8 (79.6–126.1)	*c*	*c*	12.9 (2.6–20.8)	0.50	0.66
Platelet counts, × 10^9^/L												
< 40 (*n* = 10)	14.2 (6.8–580.1)	0.80	0.10	0.9 (0–5.0)	0.52	0.28	105.7 (34.1–147.1)	0.40	0.60	6.9 (2.6–25.9)	0.32	0.47
≥ 40 (*n* = 7)	14.7 (9.8–167.0)	- 0.21	0.78	0.8 (0.6–1.8)	- 0.50	0.66	65.8 (41.4–79.6)	- 0.63	0.36	12.9 (7.5–20.8)	0.50	0.66
Relapse-risk group^e,a^												
Low/Intermediate (*n* = 11)	13.3 (6.8–580.1)	–	–	2.3 (0–5.0)	–	–	66.5 (34.1–285.1)	–	–	13.6 (3.3–25.9)	–	–
High (*n* = 6)	93.1 (19.2–167.0)	–	–	0.8 (0–1.8)	–	–	102.8 (79.6–126.1	–	–	12.9 (2.6–20.8)	–	–
Death during induction^d^												
Yes (*n* = 2)	167.0 (167.0)	–	–	0.8 (0.8)	–	–	79.6 (79.6)	–	–	20.8 (20.8)	–	–
No (*n* = 15)	13.8 (6.8–580.1)			1.76 (0–5.0)			75.9 (34.1–285.1)			7.5 (2.6–25.9)		

^a^ Nominal variable

^b^ Spearman’s R correlation considering a significance at *p* < .05 (*)

^c^ The number of data pairs are too small to stablish a correlation

^d^ Dichotomous variable

^e^ Classification according to PETHEMA-GIMEMA criteria

**Table 3:** Clinical characteristics and laboratory results at diagnosis of APL patients with or without DS

Variable at diagnosis	All patients, (*n* = 17)		DS group, (*n* = 6)				Non-DS group, (*n* = 11)			*P*-value^2^
	N.	%	Median (range)	N.	%	Median (range)	N.	%	Median (range)	
Gender										0.64
Female	10	58.8		3	50		7	63.6		
Male	7	41.2		3	50		4	36.4		
Age, years			36.25 (17.42, 72.08)			38.37 (21, 59.33)			36.25 (38.33, 72.08)	0.40
< 40	7	41.2		2	33.3		5	45.5		
≥ 40	10	58.8		4	66.7		6	54.5		
Leukocyte counts, ×10^9^/L			2.4 (0.5, 83.5)			2.0 (1.6, 48.7)			1.6 (1.2, 83.5)	0.02*
< 10	11	64.7		2	33.3		9	81.8		
≥ 10	6	35.3		4	66.7		2	18.2		
Platelet counts, ×10^9^/L			36 (5, 110)			43 (39, 71)			28 (14, 110)	0.98
< 40	10	58.8		3	50		7	63.6		
≥ 40	7	41.2		3	50		4	36.4		
Relapse-risk group^1^										0.10
Low/Intermediate	11	64.7		2	33.3		9	81.8		
High	6	35.3		4	66.7		2	18.2		
Fibrinogen (mg/dL)			152.7 (66, 400)			207.5 (77, 400)			146.7 (66, 390)	0.48
< 170	8	50		3	50		5	50		
≥ 170	8	50		3	50		5	50		
Unknown	1	–		–	–		1	–		
Death during induction										0.10
Yes	2	12.5		2	33.3		0	0		
No	15	87.5		4	66.7		11	100		

*APL* Acute promyelocytic leukemia, *DS* Differentiation syndrome

^1^Classification according to PETHEMA-GIMEMA criteria

^2^Fisher’s two-tailed exact test was used to compare categorical variables. Mann-Whitney U test was used to compare continuous variables

* Indicate values statistically significant when *P* < .05

## Discussion

The absence of IL-1β, IL-10, IL-12p70 and TNF-α expression in our cohort of patients with APL, observed in the groups with and without DS, highlights the difference in cytokine profiles of APL and non-APL AML because Turzanski et al. and Sanchez-Correa et al. have reported that IL-1β and IL-10 are detected in the plasma of patients with AML without t(15;17) and may play a role in apoptosis-resistant phenotype and clinical outcome, respectively [18, 19].

In this study, among the cytokines examined, IL-6 and IL-8 were the only ones detected on the plasma of patients with APL, but differentially modulated by treatment with ATRA over time. Similar to our results, Dubois et al. [1] demonstrated that the incubation of APL primary cells with ATRA did not modulate the IL-6 production but significantly decrease the levels of IL-8 in the supernatant. In addition, in contrast to the previous report, showing an relationship between high IL-8 serum levels and the occurrence of DS in two patients with APL [15], our data demonstrate that patients who develop or not the DS may persist with detectable low plasma concentrations of IL-8 after the first week of treatment, suggesting that the modulation of IL-8 levels in patients with APL following ATRA treatment may occur regardless of the DS development.

A key problem in the diagnostic process of DS is the lack of precise definitions of clinical criteria and biomarkers. In addition, the DS diagnosis is often challenging when the signs and symptoms attributable to DS occur in patients with APL who also develop complications such as pneumonia, cardiac toxicity, renal failure, and septic shock [6]. Considering the APL-DS patients with apparently no other complications, Montesinos et al. proposed criteria for APL-DS severity grading based on the presence of predefined signs and symptoms [20] but this classification has not been widely adopted in practice as occurred in the current study once the number of patients enrolled in the cohort did not allow a stratified analysis of the DS-group. Although the lower serum levels of IL-8 has been reported to be associated with longer survival in AML patients [13], the prognostic relevance of IL-8 in APL remains to be established in larger cohorts that accurately stratify the patients with APL for severity ranking of the DS.

### Study limitations

Limitations of the current study include the small sample size from a single center as well as the retrospective study design. In addition, cytokine measurements were achieved by using a single method.

## Conclusions

The present study demonstrated that ATRA treatment reduces the levels of IL-8 regardless of the occurrence of DS and, therefore, our findings do not support that IL-8 is a predictive biomarker for monitoring the development of the APL-DS.

## Supplementary information

**Additional file 1.** Clinical characteristics, laboratory results, and IL-6 and IL-8 levels in APL patients at D0 and during the days of treatment with ATRA and anthracyclines (D3 and D7).

## Data Availability

All data generated or analysed during this study are included in this article and in the Supplementary file 1.

## Abbreviations

AML: Acute myeloid leukemia
APL: Acute promyelocytic leukemia
ATRA: All-trans retinoic acid
DS: Differentiation syndrome
ICAM: Intercellular Adhesion Molecule
IL: Interleukin
SIRS: Systemic inflammatory response syndrome
TNF-α: Tumor necrosis factor-α
